# Insights Into the Micelle-Induced β-Hairpin-to-α-Helix Transition of a LytA-Derived Peptide by Photo-CIDNP Spectroscopy

**DOI:** 10.3390/ijms22136666

**Published:** 2021-06-22

**Authors:** M. Victoria Gomez, Margarita Ruiz-Castañeda, Philipp Nitschke, Ruth M. Gschwind, M. Angeles Jiménez

**Affiliations:** 1 IRICA, Department of Inorganic, Organic and Biochemistry, Faculty of Chemical Sciences and Technologies, Universidad de Castilla-La Mancha (UCLM), Av. Camilo José Cela 10, 13071 Ciudad Real, Spain; Margarita.RAlvaro@uclm.es; 2 Institute of Organic Chemistry, University of Regensburg, Universitätsstraße 31, 93053 Regensburg, Germany; Philipp.Nitschke@murdoch.edu.au (P.N.); ruth.gschwind@ur.de (R.M.G.); 3 Departamento de Química-Física Biológica, Instituto de Química Física Rocasolano (IQFR-CSIC), Serrano 119, 28006 Madrid, Spain

**Keywords:** photo-CIDNP, NMR spectroscopy, in situ illumination, LytA-derived peptide, tyrosine side chains, molecular motion, conformational transition

## Abstract

A choline-binding module from pneumococcal LytA autolysin, LytA_239–252,_ was reported to have a highly stable nativelike β-hairpin in aqueous solution, which turns into a stable amphipathic α-helix in the presence of micelles. Here, we aim to obtain insights into this DPC-micelle triggered β-hairpin-to-α-helix conformational transition using photo-CIDNP NMR experiments. Our results illustrate the dependency between photo-CIDNP phenomena and the light intensity in the sample volume, showing that the use of smaller-diameter (2.5 mm) NMR tubes instead of the conventional 5 mm ones enables more efficient illumination for our laser-diode light setup. Photo-CIDNP experiments reveal different solvent accessibility for the two tyrosine residues, Y249 and Y250, the latter being less accessible to the solvent. The cross-polarization effects of these two tyrosine residues of LytA_239–252_ allow for deeper insights and evidence their different behavior, showing that the Y250 aromatic side chain is involved in a stronger interaction with DPC micelles than Y249 is. These results can be interpreted in terms of the DPC micelle disrupting the aromatic stacking between W241 and Y250 present in the nativelike β-hairpin, hence initiating conversion towards the α-helix structure. Our photo-CIDNP methodology represents a powerful tool for observing residue-level information in switch peptides that is difficult to obtain by other spectroscopic techniques.

## 1. Introduction

The conformational transitions of peptides and proteins are in the crosshairs of several research fields. For example, histidine kinases undergo α-helical supercoiling to act as environmental sensors in bacteria and plants [1]. In humans, disordered regions of RIPK1 and RIPK3 kinases reversibly assemble β-sheet-rich hybrid amyloids to signal cell death [2]. Structural transitions are also a common subject in most neurodegenerative diseases, in which uncontrolled changes in the native structure of proteins trigger pathogenesis [3,4,5]. Along this line, one of the most popular examples is the conversion of the β-amyloid peptide from a soluble unstructured ensemble of conformations into a “double-horseshoe-like cross-β-sheet” [6,7] that is associated with the onset of Alzheimer’s disease. Conformational transitions are also key to emerging fields, including nanobiotechnology or de novo protein design, as they settle the basis for creating self-assembled materials of controlled geometries [8].

A common aspect to all the above findings is that a particular amino acid sequence acts as a “conformational switch”, enabling different secondary structures under distinct environments [9,10,11], i.e., pH/temperature changes or interactions with other molecules [12]. This feature makes chameleonic sequences very suitable for protein folding and design [13,14], with boundless applications ranging from the delivery of new therapeutics that block or promote these transitions [15] to the design of novel biosensors [16].

A particular case of increasing relevance is found in *pneumococcus*, the most common pathogen of the respiratory tract that causes pneumonia. These microorganisms are equipped with several surface proteins, especially choline-binding proteins (CBPs) that are essential for bacterial colonization and virulence [17], presumably with the involvement of conformational transitions. LytA autolysin is one of such proteins and is composed of seven different choline-binding repeats (CBRs) with a β-hairpin structure. The LytA_239–252_ peptide (CBR4 of LytA) converts into a stable α-helix in the presence of detergent micelles and folds back into the β-hairpin conformation by dilution up to detergent concentrations below critical micelle concentration (CMC) [18]. Because LytA_239–252_ retained both the native β-hairpin structure and the ability to bind cholines, this conformational transition might be relevant to the virulence of *pneumococcus*. In an attempt to mimic the interactions with the cell membrane, structural studies using nuclear magnetic resonance (NMR) spectroscopy and other biophysical methods were conducted to understand the interactions between α-helix and micelles [18,19]. However, detailed information on the conformational transition itself remained elusive.

Photochemically induced dynamic nuclear polarization (photo-CIDNP) is an excellent probe for scrutinizing the solvent exposure of individual tryptophan, tyrosine, or histidine amino acids in a sequence, i.e., which of those aromatic side chains are participating in an interaction (hindered from the solvent) and which are freely exposed [20]. This approach involves a spin-selective photochemical process due to the formation of a radical pair intermediate between the target molecule and a laser-excited photosensitizer dye (usually riboflavin or riboflavin derivatives) with a high triplet-state lifetime, which can lead to positive and negative enhancements of NMR signals. Since the interaction between the soluble photosensitizer and the amino acid side chain is key to the detected intensity changes, this method selectively enhances the NMR signals of tryptophan, tyrosine, and histidine residues, and can be interpreted as a measure of their exposure to the solvent [20]. This logic was previously shown in analyses of the interaction between a β-endorphin peptide and DPC micelles [21]. Interestingly, signal enhancements produced by photo-CIDNP can be transferred between nearby nuclei in a mechanism similar to the NOE effect [22]. In these cases, photo-CIDNP data are interpreted to give an idea about the mobility of a molecule, assuming that a relatively large number of conditions are surveyed [21,22]. These interesting features prompted us to use photo-CIDNP to gain insights into the micelle-triggered conformational transition observed in LytA_239–252_.

When executing photo-CIDNP experiments, the uniform illumination of the whole sample volume faces several problems: (i) magnetic-field distortions caused by an insert into the sample volume to uniformly distribute the light, (ii) high-power laser intensity is needed to observe the photo CIDNP phenomena, and (iii) the light absorption by the solution above the radiofrequency coil region that could decrease the stability of the sample and the observed signal, leading to the loss of some sensitivity and resolution [20]. The optimization of these experimental conditions is highly important because of the high dependence of light intensity in the NMR sample active volume and the photo-CIDNP signal in the NMR spectra [23,24]. Our previous experience in the development of in situ NMR illumination devices for mechanistic studies on photochemical reactions using low-power LEDs [23,25] and the use of a low-power laser-diode setup for pushing NMR sensitivity for small sample volumes [26] reveal the importance of the optimization of the photon flux in our NMR active volume for gaining all information out of photo-CIDNP phenomena.

Here, we report valuable residue-level information that affords a clear picture of the conformational transition of the LytA_239–252_ peptide from the native β-hairpin into the α-helix triggered by the presence of DPC micelles. Aromatic stacking and disruption encode the key for conformational switching. Furthermore, we describe the whole optimization process for the experimental NMR conditions for the optimal illumination of the whole sample volume. These results demonstrate the great potential of using experimental photo-CIDNP data in an optimized set of experimental conditions to determine conformational transitions of switch peptides.

## 2. Results

### 2.1. Photo-CIDNP Experiments of LytA_239–252_ Peptide in the Absence of DPC Micelles

**Figure 1:**
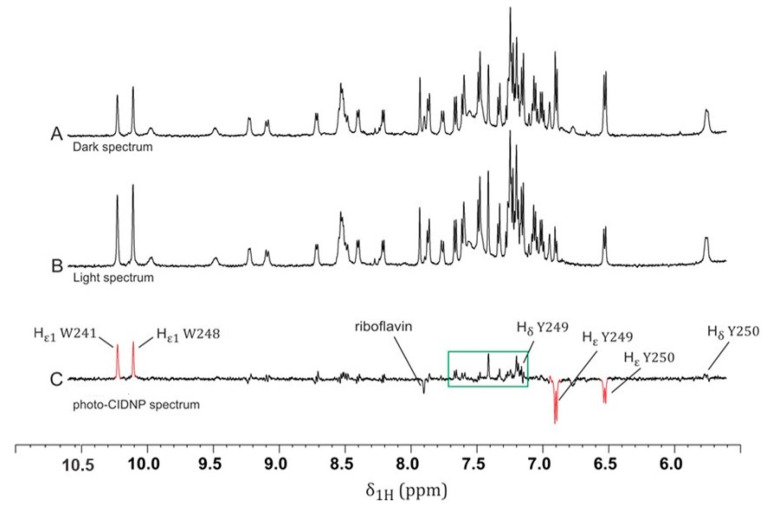
^1^H NMR spectra of 1 mM LytA_239–252_ peptide in aqueous solution (H_2_O/D_2_O 9:1 *v*/*v*): (**A**) dark spectrum; (**B**) light spectrum; (**C**) photo-CIDNP spectrum (B–A). Method A was followed for the acquisition of the NMR experiments (see Section 4). Peptide irradiation in the presence of riboflavin led to the enhancement of or reduction in different signals, particularly the Hε1 protons of tryptophan and Hε protons of tyrosine residues. CIDNP spectrum (**C**) (“light-minus-dark” representation; red signals) shows the net effect of these intensity changes. The boxed region (green) includes the aromatic signals of both W241 and W248 residues affected by photo-CIDNP (see Figure S1A for expansion). We could identify the photo-CIDNP signals of riboflavin (7.8 ppm).

Figure 1A shows the ^1^H NMR spectrum of the aromatic region of the LytA_239–252_ peptide, which we refer to as the “dark” spectrum. Upon illumination, several changes became apparent in the “light” spectrum (Figure 1B). These changes could be visualized by comparing the irradiated (“light”, Figure 1B) and nonirradiated (“dark”, Figure 1A) spectra by using a “light-minus-dark” difference spectrum (Figure 1C), where it is possible to filter out photo-CIDNP effects from the signals of unaffected residues. These photo-CIDNP effects are shown as intensity enhancements as absorptive (positive) or emissive (negative) signals in certain nuclei of residues that were exposed to the solvent.

Among all polarized signals, four strongly enhanced peaks are highlighted in Figure 1C (red)— namely, two absorptive (positive) peaks at 10.21 and 10.11 ppm corresponding to the indole protons (Hε1) of the tryptophan residues (W241 and W248), respectively, and two emissive (negative) peaks at 6.91 and 6.54 ppm from the ε-protons (Hε) of tyrosine residues (Y249 and Y250), respectively. In addition, some other, weak enhancements appeared in the aromatic region spanning 7.0–7.7 ppm (see boxed peaks in Figure 1C), corresponding to the aromatic protons of W241 and W248 and the Hδ of Y249. Figure S1 shows an expansion of this region with annotated peaks identifying each NMR signal. All these absorptive and emissive enhancements are in accordance with the characteristic hyperpolarization patterns of tryptophan and tyrosine residues [20].

**Figure 2:**
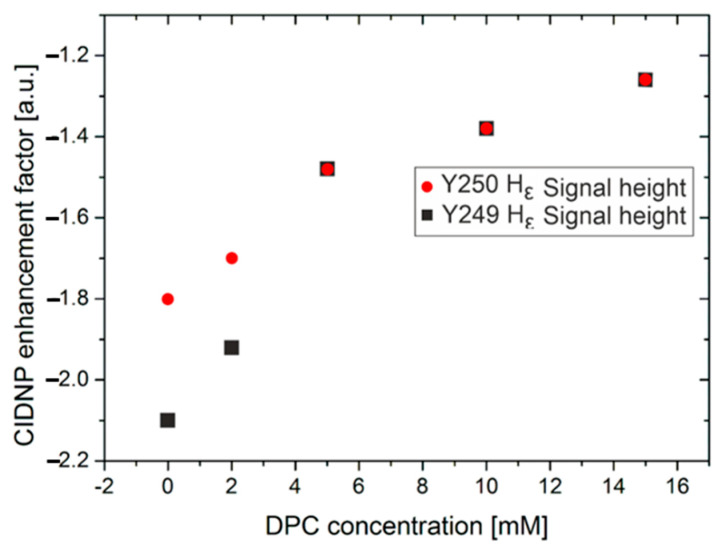
Photo-CIDNP enhancement of Hε signals of Tyr side chains (Y249 and Y250) as a function of DPC concentration. Signal height was used instead of integrals because Y249 is always partially overlapped with other signals at most DPC concentrations, resulting in wrong photo-CIDNP enhancement factors for the integration approach. However, at least one of the spins of the doublet of Y249 was free of overlap (at least for 0–15 mM of DPC) and could be therefore used for the determination of the photo-CIDNP enhancement factors. In the presence of 2 mM of DPC, the photo-CIDNP enhancement factor of the epsilon proton of Y249 varied by 8.6%, while the epsilon proton of Y250 varied by 5.5%, illustrating the higher accessibility of the former to the solvent.

The Hε1 proton signals in both tryptophan residues (W241 and W248) were enhanced to a similar extent, although the intensity of W241 seemed to be slightly lower than that of W248; in fact, the photo-CIDNP enhancement factors were 2.2 (integral value of 1.0) and 2.3 (integral value of 1.2), respectively. However, the two tyrosine residues, Y249 and Y250, exhibited strikingly distinct behaviors (Figure 1C). The enhancement in Y249 (CIDNP enhancement factor −2.1; Figure 2; 0 mM DPC) was larger than that in Y250 (CIDNP enhancement factor −1.8, Figure 2; 0 mM DPC). These changes were due to different solvent exposure and were thereby due to accessibility to the flavin. In other words, Y249 was much more exposed to the solvent than Y250 was; as mentioned above, W241 was slightly less exposed than W248 [27] was. This result correlates with visual inspections of the 3D structure of the LytA_239–252_ peptide in β-hairpin conformation (Figure S2) [18], which showed that the aromatic side chains of W241 and Y250 were stacked against each other. This tight packing rendered the aromatic protons in W241 and Y250 less accessible to the flavin, thus explaining the lower signal enhancements observed in the photo-CIDNP spectrum (Figure 1C) for both residues in comparison with W248 and Y249. The effect on tryptophan residues was less notable, probably due to the existence of other degenerate exchange–secondary reactions [28] or chemical-exchange reactions with the water of the media, as they corresponded to labile NH indole protons. [29] These observations highlight the potential of photo-CIDNP as an appropriate tool for selectively monitoring the environment of aromatic residues [27].

### 2.2. Photo-CIDNP Experiments of LytA_239–252_ Peptide in the Presence of DPC Micelles

In the presence of increasing DPC micelle concentration in the range of 0–30 mM, (critical micelle concentration (CMC) ~1.2 mM for our experimental conditions) [18], the LytA_239–252_ peptide underwent a β-hairpin-to-α-helix conformational transition. The structure of this peptide in such a helical conformation and the residues involved in the interaction with the micelles were characterized using NMR spectroscopy [18]. Particularly, tryptophan residues were the most involved in contacts between micelle and α-helix, orienting the helical hydrophobic face towards the micelle core, whereas its hydrophilic face (Lys243 and Lys247) lay onto the micelle surface. These previous reports, however, did not provide information about tyrosine residues [18] and were not able to differentiate between analogous residues that behaved distinctly, i.e., exposed to or buried in the solvent. Here, photo-CIDNP experiments were carried out in the presence of increasing DPC concentrations to provide additional information about the interaction between peptide and micelle, especially when focusing on the tyrosine residues.

**Figure 3:**
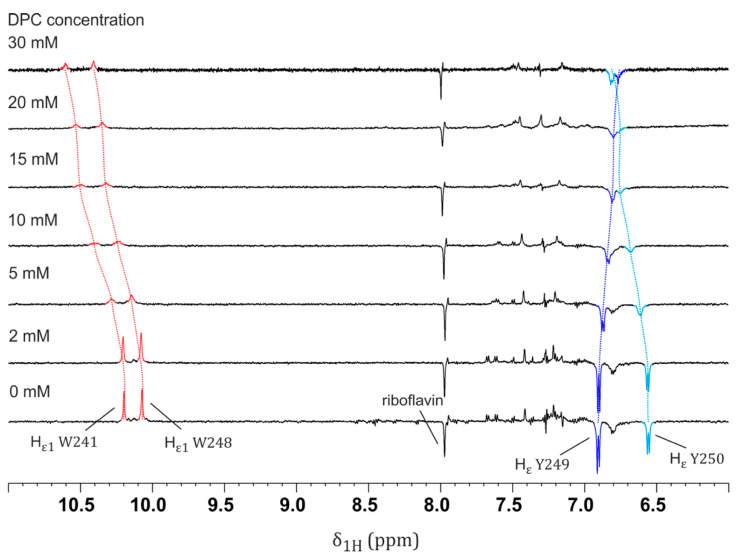
Photo-CIDNP (“light-minus-dark” representation) NMR spectra for DPC micelle titration (from 0 to 30 mM) of 1 mM LytA_239–252_ peptide. Method A was followed for the acquisition of NMR experiments (see Section 4). The increase in micelle concentration led to the chemical shifting and line broadening of NMR signals, which are affected by the presence of micelles, especially those corresponding to Hε1 of W241 and Hε of Y250. Lower CIDNP intensities obtained when increasing micelle concentration were not due to flavin–micelle interactions, since unlike peptide NMR signals, the one corresponding to flavin (8.0 ppm, emissive) is not affected by the presence of micelles at all.

Figure 3 illustrates the photo-CIDNP of the two Hε proton signals from Y249 and Y250 as well as the Hε1 proton of both W241 and W248, with DPC concentrations in the range of 0–30 mM. Here, different residues should not be compared (i.e., pair W241/W248 with pair Y249/Y250) but rather W241 vs W248 and Y249 vs Y250. First, an overall drop in photo-CIDNP intensity for all signals could be observed for increasing concentrations of DPC. This was due to the transition of the peptide from the β-hairpin to the α-helix conformation, increasing interaction with DPC resulting in an overall reduced solvent exposure, and hence smaller photo-CIDNP effects. However, the behavior of the two tyrosine residues is of special interest. As mentioned above, the two tyrosine residues showed different photo-CIDNP enhancement factors without the presence of DPC (Y249—2.1; Y250—1.8; see Figure 2 and Figure 3). However, with increasing concentration of DPC, the photo-CIDNP enhancement factors of Y249 and Y250 started to align and were similar at DPC concentrations above 5 mM (Figure 2). The photo-CIDNP enhancement factors of both tyrosine residues also seemed to align for DPC concentrations in the range of 20–30 mM, but it was difficult to exactly determine the enhancement due to the heavy signal overlap at these DPC concentrations (Figure 3). This indicates that Y249 was more accessible to the flavin in the β-hairpin than Y250 was, but in the α-helix, they were both equally accessible. In contrast to tyrosine residues, tryptophane residues W241 and 248 showed almost identical photo-CIDNP enhancement factors from the start at 0 mM DPC (W241 2.2; W248 2.3); upon increasing DPC concentration, their photo-CIDNP enhancement factors stayed rather constant with respect to each other. This was probably due to the poor signal-to-noise ratio of the photo-CIDNP effects for W241 and W248 at higher DPC concentrations (≥5 mM), preventing the clear detection of small enhancement changes, and their chemical exchange with water, as mentioned above. However, they also showed an overall drop in photo-CIDNP intensity due to the peptide transition. This was in agreement with the side-chain exposure of these residues in the 3D helical conformation of this peptide (see Figure S3). [18] The above observations might be interpreted in terms of all W241, W248, Y249, and Y250 side chains participating in the interaction with the micelle, as shown by the intermolecular NOEs observed at 30 mM DPC (deuterated DPC-d38/nondeuterated DPC 1:1) [19] and further corroborated by recording selective 1D ^1^H,^1^H-NOESY and 2D ^1^H,^1^H-NOESY experiments. On their basis, we were able to confirm that the interaction between peptide and micelle was mainly mediated by the aromatic side chains of both tyrosine (Y249 and Y250) and tryptophan (W241 and 248) residues (Figure S4A–F). Attempts to monitor the changes induced by DPC looking at the photo-CIDNP signals of W241 and W249 (Hα) were unsuccessful due to sensibility problems (low S/N) and line broadening.

Notwithstanding the valuable insights into the micelle–helix interaction mode afforded by these data, this “static picture” does not provide information on the conformational change process. Considering the role of LytA in infection, and more broadly the intriguing “chameleonic” nature of this and other peptide sequences, understanding how external conditions (i.e., extrinsic to the sequence) promote structural changes is of great interest. This is why we performed titrations with increasing amounts of DPC (as mentioned above) in order to determine the maximal DPC concentration at which the LytA_239–252_ peptide mainly existed as a β-hairpin, which helped us to investigate the earliest moments of the conformational transition. As shown in Figure 3, in the presence of 5 mM of DPC micelles, the NMR signals corresponding to W241, W248, Y249, and Y250 were broadened and slightly shifted. This is evidence of a strong interaction between micelle and peptide [21,30]. Whereas this broadening hampers photo-CIDNP studies in the presence of DPC concentrations between 5 and 30 mM (see Figure 2), using 2 mM DPC is enough for understanding the conformational change by photo-CIDNP experiments. That the peptide interacts with the micelle is demonstrated by the presence of numerous intermolecular NOEs at 30 mM DPC (Figure S4A–F) [19].

At DPC concentrations below 5 mM, the β-hairpin is still present, as gauged by the comparison of the photo-CIDNP spectrum at 0 mM DPC (100% β-hairpin, Figure 3 bottom spectrum) and at 30 mM DPC (approximately 71% α-helix in fast equilibrium with β-hairpin [19], Figure 3 top spectrum). Interestingly, when moving from 2 to 5 mM, and then to 30 mM of DPC micelles, larger intensity changes were observed for the Hε proton signals of Y250 (6.54 ppm) with respect to that of Y249 (6.91 ppm) (Figure 2 and Figure S5). This observation is of high relevance to the conformational transition, as the aromatic side chain of Y250 packs against the indole moiety of W241 in the β-hairpin structure (Figure S2). Because both tyrosine residues are equally exposed in the α-helix, as gauged from the similar photo-CIDNP enhancement factors of their Hε protons at DPC concentrations of 5 mM and above (Figure 2), and as revealed by its 3D structure (Figure S3), our data suggest that DPC micelles might disrupt the aromatic stacking between edging side chains W241 and Y250 that hold the β-hairpin folded.

### 2.3. Optimization of Light Intensity in NMR Active Volume to Observe Cross-Polarization Effects

Previously [26,31], we reported the use of a laser-diode illumination device for NMR signal enhancement with unprecedented limits, illustrating the advantages of the combination of photo-CIDNP and small NMR active volumes due to the dependency between the signal enhancement observed in the photo-CIDNP spectrum and the photon flux in the sample [23,32]. However, our aim here was using this light setup for photo-CIDNP experiments with a different application, that is, as a tool for investigating the different mobility of two tyrosine residues in LytA_239–252_.

In this sense, an exhaustive optimization of experimental NMR conditions and parameters is needed to gain all information provided by the photo-CIDNP experiments, particularly to observe cross-polarization phenomena that render differences between the mobility of two tyrosine residues, Y249 and Y250, to understand the conformational change of the peptide in the presence of micelle.

Two different NMR tube diameters, 5 mm (500 µL) and 2.5 mm (200 µL) were tested. The use of the laser-diode illumination device previously reported by us [26,33] enabled the optimal and uniform illumination of the NMR sample contained in the measuring coils of the spectrometer only when 2.5 mm tubes were used. This was revealed by the primary polarization of the Hε of Y249 and Y250 in LytA_239–252_, which showed up in the NMR spectrum as null or negative peaks in accordance with the expected patterns for a photo-CIDNP spectrum of tyrosine [22] for the 2.5 mm tube experiment, in contrast to their signals for 5 mm NMR tubes, where both appeared as positive peaks (Figure S6).

Next, light intensity within the active volume of the 2.5 mm NMR tubes needed to be optimized, focusing on avoiding signal distortion, optimal signal-to-noise ratio (S/N), and the expected negative patterns for Hε, as mentioned above. Hence, different light pulses and light current intensities were tested. In our previous research, our illumination device was continuously irradiating the sample; however, light pulses were needed to carry out this application. Hence, the laser diode was switched by a signal from the time control unit of the spectrometer. For a current intensity value of 600 mA (corresponding to 200 mW out of the optical fiber), the duration of the light pulse was varied to 0.8, 1.5, and 3 s. Higher values than 3 s induced signal distortion. Figure S7a shows the highest signal enhancement for the Hε of Y250 when a light pulse of 3 s was used in the absence of DPC in the reaction media. However, the selected light pulse for all experiments of the next section turned out to be only 0.8 s because, in the presence of DPC, light pulses of 1.5 or even 3 s resulted in lower S/N in the NMR spectra (Figure S7b), presumably due to magnetic-field inhomogeneities because of the potential heating of the sample. For comparison, the light-pulse duration should be the same in the absence and presence of DPC. Figure S8 also illustrates the importance of irradiating the sample with enough light intensity as can be observed with the expected patterns for Hε of tyrosine residues, only visible when 600 mA was used. Thus, 600 mA, 0.8 s of light pulse, and 2.5 mm NMR tubes were the optimal values when carrying out the next experiments (Method B, see Section 4).

### 2.4. Cross-Polarization Signals for Hδ of Y249 and Y250

The strong enhancements shown by the Hε protons of tyrosine and Hε1 protons of tryptophan (see Section 2.1 and Section 2.2), which display hyperfine coupling interactions within the radical pair formed between the amino acid and the flavin upon irradiation [20,34], are denoted as “primary polarizations” [22]. These can be transferred to nearby nuclei through dipolar cross-relaxation. These pumped NOEs, which are significantly sensitive to the rate of molecular tumbling, were named “cross-polarizations” [22]. The extent of cross-polarization in a molecule is a sensitive function of molecular tumbling and internal motion. Remarkably, cross-polarizations can occur with either phase inversion or phase retention with respect to that of the primary polarization [35]. In larger molecules (proteins), polarization is transferred with phase retention, but in small molecules (amino acids), transfer occurs with phase inversion [21,22]. In previous studies of peptides in the presence of micelles, the altered dynamics from residues involved in the interactions with the micelles were captured by both the CIDNP intensities of primary polarizations and cross-relaxation effects, and they were analyzed by means of theoretical models [21]. Using a theoretical model, Hore and coworkers [21] simulated the evolution of the CIDNP intensity with time for a Tyr residue of a 31-residue peptide tumbling at different rates, finding that the effect (phase retention or phase inversion) observed in the Hδ protons of tyrosine (cross-polarization effects) upon light irradiation depends on the mobility of the tyrosine residue. Cross-polarization effects occurs throughout a dipolar relaxation mechanism analogous to the NOE effect [35]. As a consequence of this phenomenon, and most importantly, the peak sign of the Hδ proton of a Tyr in a free peptide (fast tumbling) remains positive (the cross-polarization effect occurs with phase inversion), while for a micelle-interacting Tyr residue (slow-tumbling, restricted molecular motion), the peak sign for delta proton changes (cross-polarization occurs with phase retention) and both signals appear as negative peaks [21].

These previous data prompted us to examine the cross-polarization effects for the Tyr residues of the LytA_239–252_ peptide. Interestingly, the Hδ resonances of the two Tyr residues behaved differently, as shown in Table 1. In the presence of micelle (2 mM, above CMC), a clear sign inversion occurred for the Hδ of Y250 as it went from positive (+1.78) to negative values (−1.33) (phase retention) upon increasing micelle concentration (from 0 to 2 mM), while the observed signal for the Hδ of Y249 remained positive (phase inversion) (see Table 1). These results are in accordance with Hore and coworkers [21], and therefore show that Y250 is involved in a stronger interaction with DPC micelles than it is with Y249.

Hore et al. [21] found differential influence of DPC binding on two tyrosine residues in β-endorphin, observing stronger immobilization for only one of the tyrosine residues when calculating their individual correlation time. Continuing with the approach reported by Hore et al. [21], where the molecular motion for the different residues was estimated representing ρ = Int(Hδ)/Int(Hε) [21] vs correlation time, and comparing our experimental data (ρ) with their model due to a similar case (DPC and short peptide), we could predict a noticeable difference in the correlation time for Y249 and Y250. Hence, the different values for ρ_Y250_ when moving from 0 to 2 mM of DPC (see Table 2) in comparison with ρ_Y249_ indicated that we could expect a very different value for the correlation time of Y250. This rough estimation of different molecular motions for the two tyrosine residues should only be considered as relative changes within a certain tyrosine residue, as ρ_Y249_ does not significantly change in the presence of micelles, in contrast to Y250, which went from a negative (–0.21) to a positive value (+0.15).

## 3. Discussion

Here, the potential of the photo-CIDNP NMR technique is outlined for the identification of solvent-accessible aromatic residues within a peptide sequence to understand the interaction between the LytA_239–252_ peptide and DPC micelles. In our case of study, photo-CIDNP experiments revealed that Tyr residues from the LytA_239–252_ peptide were distinctly exposed to the solvent; more precisely, Y250 was less exposed to the solvent in the aqueous solution due to aromatic stacking than Y249 was and became more hindered in the presence of increasing DPC amounts as illustrated by the lower signal enhancement for the primary polarizations (looking at the Hε) compared with Y249.

Focused on gaining insight into the different behaviors of the two Tyr residues, Y249 and Y250, and to understand whether they interacted with the micelle differently, we aimed to observe the cross-polarization effects (looking at Hδ) due to its dependency on molecular motion [21]. Interestingly, the Hδ protons for both Y250 and Y249 showed a different sign in the presence of the micelle. Phase inversion (positive sign) was observed for the Hδ of Y249, in contrast to a clear phase retention (negative sign) that occurred for the Hδ of Y250 when the concentration of micelle was increased from 0 to 2 mM. As reported previously, this phase retention [22] could be attributed to an alteration in the molecular motion that might be caused by interaction with a micelle [21]. Therefore, the different sign of photo-CIDNP signals for the Hδ of Y249 and Y250 allowed us to conclude that Y250 is involved in a stronger interaction with DPC micelles than Y249 is; then, the molecular motion of Y250 increases to such an extent that the polarization transfer from Hε to Hδ causes the phase inversion of the H_δ_ signal [21].

The use of 5 mm NMR could not provide efficient and uniform light transmission over the whole sample volume in our experimental conditions due to optical-density problems. The use of smaller 2.5 mm diameter tubes is required to achieve the optimal primary polarization of Hε, resulting in the observed differences for the cross-polarization effect of the Hδ. In addition, due to the high dependency between the signal intensity in the photo-CIDNP spectrum and the light intensity in the active sample volume [23,24], an exhaustive optimization of the experimental parameters (light pulses, light intensity, NMR pulse sequence) is required to reach the maximal signal enhancement for the Hε of the two tyrosine residues, Y249 and Y250, and to gain the dynamic information out of the photo-CIDNP experiments.

Considering that the freedom of some residues can be altered through molecular interactions and that this phenomenon can be quantified thanks to individual rotational correlation time values as previously reported by Hore et al. [21], the comparison of our experimental data with their reported models clearly confirmed the difference in mobility for the two tyrosine residues. By measuring the experimental CIDNP intensity values (integrals from the spectra) for the Hδ and Hε protons of both Tyr residues, a rough estimation of their different rotational correlation time was extracted using the reported models as reference for a DPC micelle concentration range in which the peptide remained as a β-hairpin (0 to 2 mM). This observation demonstrated the different behaviors of the two Tyr residues and corroborated that Y250 (stacked in the β-hairpin) was substantially more affected by the interaction with DPC micelles than was the other tyrosine residue, Y249 (not involved in intraresidue interactions), illustrating that the two tyrosine residues tumble at very different rates in the presence of DPC.

All these data about the difference behavior of both Tyr residues can be used to understand the conformational change of LytA_239–252_ in the presence of a DPC micelle, which could be interpreted in terms of the DPC micelle disrupting the aromatic stacking between W241 and Y250, as the latter is involved in a hydrophobic interaction with W241 that also justifies its lower solvent exposure to the solvent and thereby its lower photo-CIDNP effects.

In conclusion, this work represents a new example where photo-CIDNP proved to be a useful tool for studying the architecture of biological macromolecules, particularly for investigating structural changes in peptides because of molecular interactions. Photo-CIDNP provides experimental data about the solvent accessibility of Tyr side chains, which are difficult to obtain by other spectroscopic techniques. For instance, fluorescence gives information about tryptophan sidechains [18] but not Tyr sidechains. On the whole, our results open new avenues for interpreting conformational transitions, such as the case of switch [9,10,11] and self-associating peptides [36] and render valuable information for combating structural changes that cause virulence and pathogenesis.

## 4. Materials and Methods

### 4.1. Light Sources

#### 4.1.1. Light-Emitting Diode (LED)

The high-power LED was controlled by an electronic circuit that consisted of a power supply, the LED (Cree XT-E LED, US, with a center wavelength of 455 nm and 500 mW optical output power), a potentiometer to regulate the current through the LEDs, and a transistor switched by a TTL signal from the time control unit of the spectrometer. With this circuit, the LED could be directly switched on and off by the spectrometer. The setup enabled the optimal and uniform illumination of the NMR sample in the measuring coil of the spectrometer, as previously reported by us [23].

The claddings of the optical fiber tip were stripped off, the main part of the fiber was masked, and the 21 mm at the tip of the fiber was sandblasted to roughen its surface and to thus make it emit light over its whole range instead of just from its tip. Silicium carbide 180 was used as the abrasive material [23].

#### 4.1.2. Laser Diode

The low-power light source used for the experiments consisted of a laser diode (Thorlabs, L450P1600MM) emitting at 450 nm and operating at 1.6 W output power. The optical power was lowered to the milliwatt range by using a potentiometer that regulated the current through the diode, enabling the selection of the minimal current to achieve maximal signal enhancement to avoid signal distortion due to sample overheating. A photometer was used to monitor the desired power from the light beam coming out of a BFH optical fiber with 1000 μm diameter, purchased from Thorlabs (Germany). This current controller (Thorlabs, LDC 240C) could be switched on and off to regulate the duration and intensity of the light pulse either manually (continuous irradiation) or in a pulsed manner by a TTL signal directly from the spectrometer. A temperature controller (Thorlabs, TED 200C) was also used to ensure no variation in output light power of the current controller (LDC, 240C) for a certain current value [26].

The claddings of the optical fiber tip were stripped off, the main part of the fiber was masked, and the 21 mm at the tip of the fiber was sandblasted to roughen its surface, thus making it emit light over its whole range instead of just from its tip. Silicium carbide 180 was used as the abrasive material [23].

### 4.2. NMR Sample Preparation

An amount of 1 mg of LytA_239–252_ (TGWKKIADKWYYFN) (DG peptides Co., Ltd., Hangzhou, China; http://www.dgpeptides.com/en, accessed on 5 November 2019) was used. A mixture of H_2_O/D_2_O 9:1 ratio by volume (99.9%, Cambridge Isotope Laboratories (MA, USA) and Merck) was used for Method A, and D_2_O was used for Method B, as we were not interested in observing the Hε of W241,248. Riboflavin (Sigma-Aldrich, St. Louis, MO, USA) was used as the photosensitizer. Riboflavin required the use of DMSO to be dissolved. pH was adjusted to 3.0 with the addition of a diluted chlorohydric acid (HCl) solution. Hydrogen peroxide (H_2_O_2_) was used for riboflavin reoxidation during the dark experiments in Method A. Increasing concentrations of n-dodecylphosphocholine (DPC) (Avanti Polar Lipids, AL, USA) up to 30 mM were employed for the conformational change of the peptide; 1 mM of 4,4-dimethyl-4-silapentane-1-sulfonic acid (DSS) or 3-(trimethylsilyl)propionic-2,2,3,3-d4 acid (TSP) was employed as the internal NMR standard.

### 4.3. NMR Experiments

Different approaches were followed depending on the NMR spectrometer and the employed light source.

#### 4.3.1. Method A

NMR spectra were recorded on a Bruker Avance 600 MHz spectrometer with a 5 mm broadband triple resonance z-gradient probe. A temperature of 308 K was controlled by a Bruker BVTE 3000 unit. Spectra were acquired for 1 mM of peptide and 0.8 mM of riboflavin at 308 K and pH = 3.0, with 1 s of illumination (t_L_) at 1290 mV and a delay between the light pulse and the 90° radiofrequency pulse (t_D_) of 10 ms. A relaxation delay of 30 s was chosen to give time to the system to return to its equilibrium. As a light source, a high-power LED-illumination device was employed [23].

For the photo-CIDNP experiments, the subsequent procedure was followed: starting with a ^1^H NMR nonilluminated spectrum of 1 scan, ^1^H NMR spectra (all of them of 1 scan) were collected, and the light was switched on and off between two consecutive spectra. Dark and light spectra were independently accumulated in an interleaved manner and were summed up—that is, instead of performing a single experiment with several scans, the accumulation of several single scans was carried out, thus arising to the same result and preventing flavin photodegradation. CIDNP spectra were obtained with the subtraction of “accumulated” light and dark spectra.

#### 4.3.2. Method B

The NMR spectra were recorded on a Varian INOVA 500 MHz NMR spectrometer equipped with a gradient amplifier and a four-nucleus 5 mm ^1^H{^15^N-^31^P}PFG high-field indirect detection probe. NMR tubes with an external diameter of 2.5 mm (purchased from Wilmad, NJ, USA) were used for the more efficient and uniform illumination of the whole sample volume [26]. Spectra were acquired for 2 mM of peptide and 0.5 mM of riboflavin at 298 K and pH = 3.0, with 0.8 s of illumination (t_L_) at 600 mA and a delay between light pulse and 90° radiofrequency pulse (t_D_) of 100 ms. The tip of the fiber was sandblasted to roughen its surface, and thus make it emit light over the whole range instead of just from its tip, using silicium carbide 180 as the abrasive material. Higher peptide concentration in comparison with that in Method A was selected to better observe the NMR signals sensitive to the CIDNP effect, as the employed NMR probe was designed for 5 mm NMR tubes. As a light source, a low-power laser diode device was employed [26]. This method involved the use of lower active volumes for the more efficient light transmission needed to achieve the higher polarization of the Hε of Y249 and Y250 with the main aim of observing the cross-polarization phenomena with sufficient signal-to-noise ratio.

All experimental data were processed with standard MestreNova 14.1 (Spain) software. Assignments were made by a complete standard set of 1D and 2D NMR experiments consisting of ^1^H,^13^C HSQC and ^1^H,^13^C HMBC spectra. Chemical shifts were referenced to the ^1^H chemical shift of DSS or TSP at 0.00 ppm.

## Figures and Tables

**Table 1:** Photo-CIDNP NMR intensity (calculated from integral values shown by Figures S9 and S10) of Y249 and Y250 for Hδ from LytA_239–252_ peptide in aqueous solution (D_2_O) for 0 and 2 mM DPC micelle concentration (298 K, pH = 3.0). Method B was followed (see Section 4).

DPC	Hδ Y250	Hδ Y249
0 mM	+1.78	+9.55
2 mM	−1.33	+6.65

**Table 2:** ρ = integral (Hδ)/integral (Hε) for Y249 and Y250 residues (calculated from integral values shown by Figures S9 and S10) of LytA_239–252_ peptide.

DPC	ρ Y249	ρ Y250
0 mM	−0.25	−0.21
2 mM	−0.22	+0.15

## Supplementary Materials

The following are available online at https://www.mdpi.com/article/10.3390/ijms22136666/s1. Figure S1: Photo-CIDNP NMR spectrum of aromatic region of the LytA_239-252_ peptide. Figure S2: 3D structure of LytA_239–252_ peptide in β-hairpin conformation. Figure S3: 3D structure of LytA_239–252_ peptide in α-helix conformation. Figure S4A: DPC schematics. Figure S4B–F: NOESY experiments. Figure S5: Photo-CIDNP NMR spectra for DPC micelle titration of LytA_239–252_ peptide for epsilon protons of Y249 and Y250. Figure S6: Optimization of NMR tube diameter. Figure S7: Optimization of light pulse duration. Figure S8: Optimization of light intensity for optimized light pulse value. Figure S9: Expansion of ^1^H-NMR spectra for 2 mM LyA_239–252_ for dark (top) and light (bottom) conditions in the absence of micelle and in 2.5 mm NMR tubes. Figure S10: Expansion of the ^1^H-NMR spectra for 2 mM LyA_239–252_ for dark (top) and light (bottom) conditions in the presence of micelle (2 mM).
