# Supplementary material for: Insights Into the Micelle-Induced β-Hairpin-to-α-Helix Transition of a LytA-Derived Peptide by Photo-CIDNP Spectroscopy
Source: Int J Mol Sci. 2021 Jun 22;22(13):6666. doi: 10.3390/ijms22136666 (PMC8268221; doi:10.3390/ijms22136666)
Supplement: Supplementary file 1 [file ijms-22-06666-s001.zip › ijms-1195087-supplementary.pdf]

SUPPORTING INFORMATION

Insights into the micelle-induced β-hairpin-to-α-helix transition of a LytA-derived peptide by Photo-CIDNP spectroscopy.

M. Victoria Gomez,[1,*] Margarita Ruiz-Castañeda,[1] Philipp Nitschke,[2] Ruth M. Gschwind,[2] M. Angeles Jiménez.[3, *]

[1] Affiliation 1; IRICA, Department of Inorganic, Organic and Biochemistry, Faculty of Chemical Sciences and Technologies, Universidad de Castilla-La Mancha (UCLM), Av. Camilo José Cela 10, 13071 Ciudad Real, Spain. M.V.G: Mariavictoria.gomez@uclm.es; M. R. Margarita.RAlvaro@uclm.es.

[2] Affiliation 2; Institute of Organic Chemistry, University of Regensburg, Universitätsstraße 31, 93053 Regensburg, Germany. R.M.G: ruth.gschwind@ur.de; P.N: present address: Australian National Phenome Centre, Health Futures Institute, Murdoch University, Perth, WA 6150, Australia; Philipp.Nitschke@murdoch.edu.au;

[3.] Affiliation 3; Departamento de Química-Física Biológica, Instituto de Química Física Rocasolano (IQFR-CSIC), Serrano 119, 28006, Madrid, Spain majimenez@iqfr.csic.es.

* Correspondence: M. V. G: mariavictoria.gomez@uclm.es; M.A.J.: majimenez@iqfr.csic.es

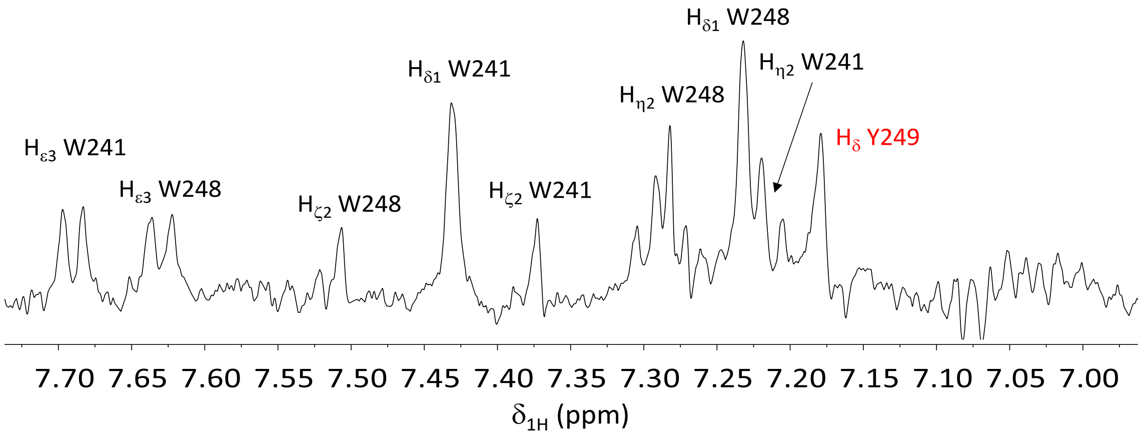

Figure S1. Photo-CIDNP NMR spectrum of the aromatic region of the LytA$_{239\text{-}252}$ peptide. The spectral region which is boxed in green in Figure 1, panel C, is expanded here. Aromatic protons from Trp side chains (W241 and W248) are labeled in the photo-CIDNP spectrum (see **Figure 1**). A signal from a tyrosine residue (Hδ from Y249, labeled in red) also appears in this region. For the nomenclature, see M. A. Jimenez and col. in Biopolymers: Peptide Science 94, 779-790 (2010) doi: 10.1002/bip.21436.

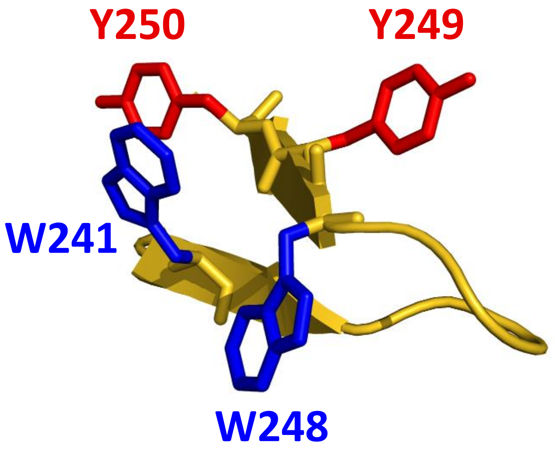

Figure S2. 3D structure of LytA$_{239\text{-}252}$ peptide in the β-hairpin conformation. The backbone trace is shown in cartoon representation (gold colour). Side chains of aromatic residues are depicted as sticks; those of Trp residues (W241 and W248) are shown in blue and those of Tyr residues (Y249 and Y250) in red.[18]

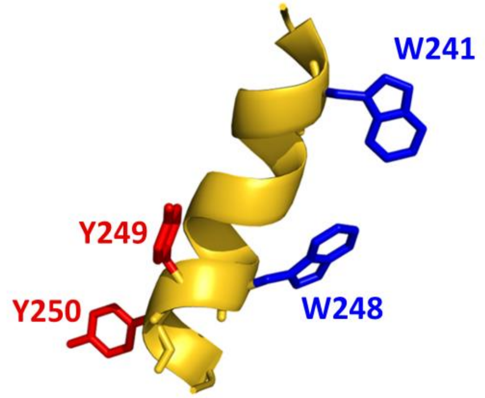

Figure S3. 3D structure of LytA$_{239\text{-}252}$ peptide in the α-helix conformation. The colour code is that used in **Figure S1B** (β-hairpin conformation): backbone trace in cartoon representation (gold), and Trp (W241 and W248, in blue) and Tyr side chains (Y249 and Y250, in red) shown as sticks. [18]

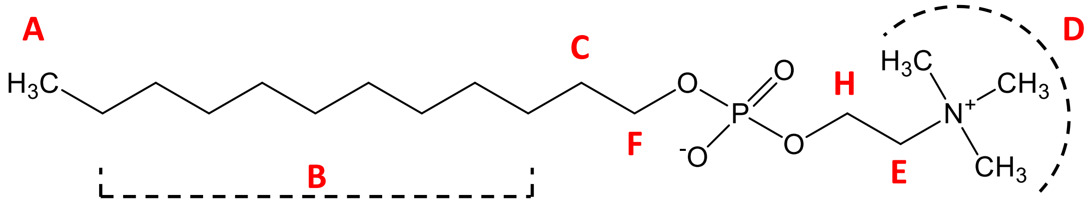

n-dodecylphosphocholine

Figure S4A. Schematics of DPC

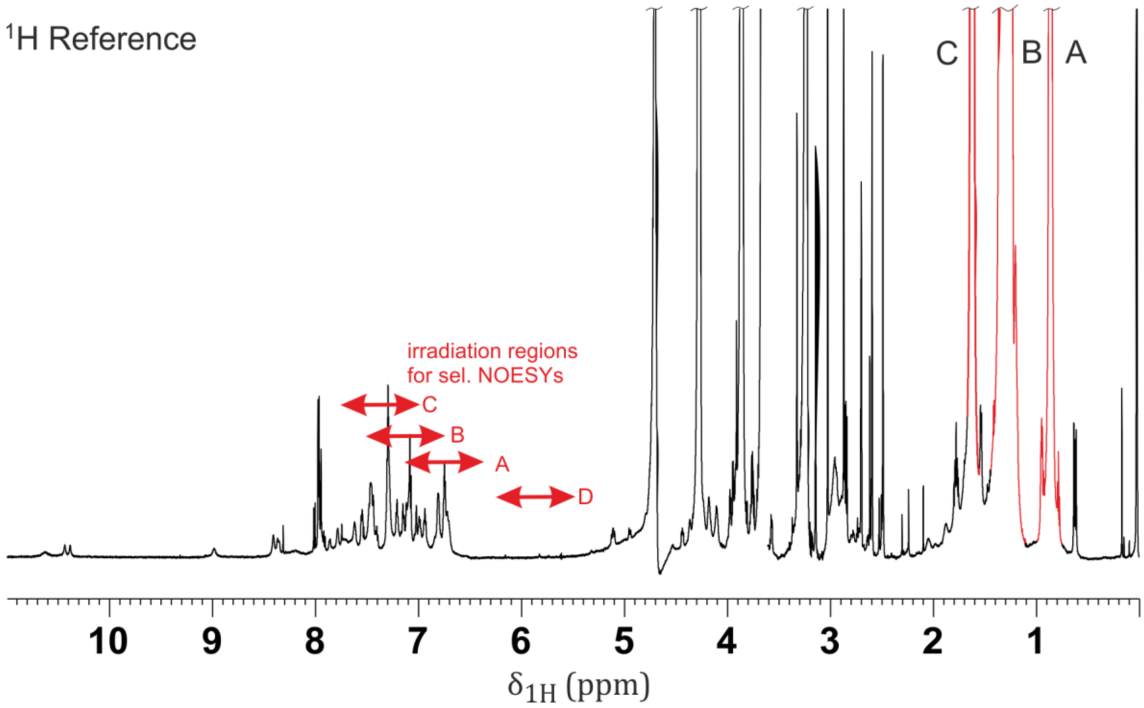

Figure S4B. 1H Reference

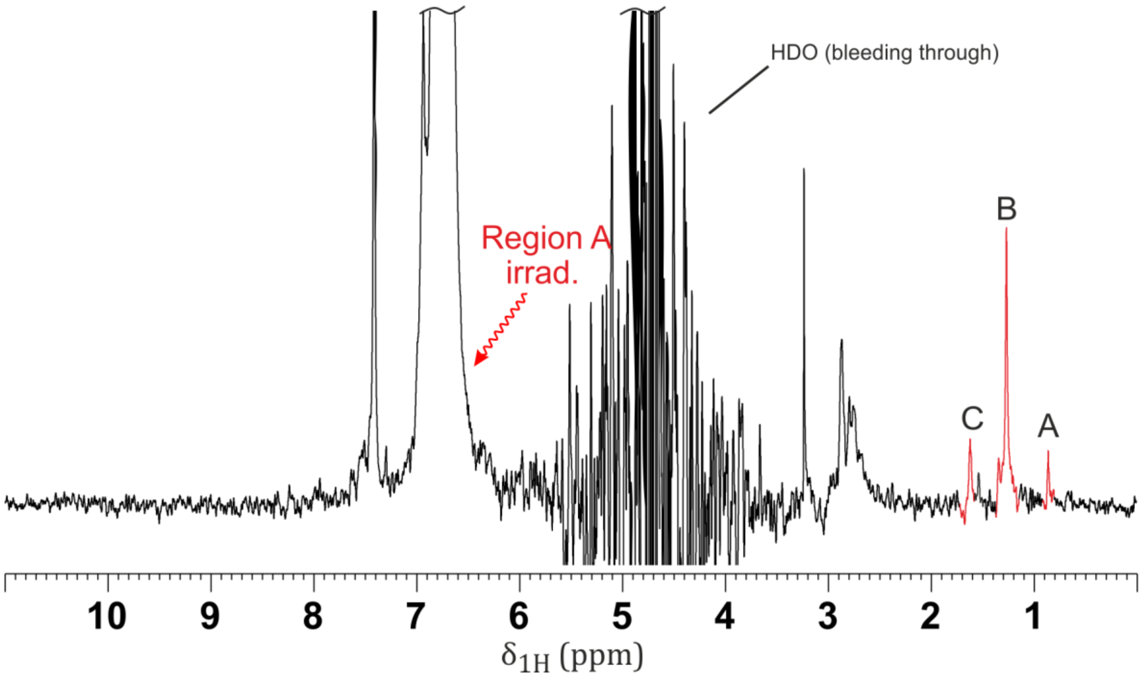

Figure S4C. Selective NOESY

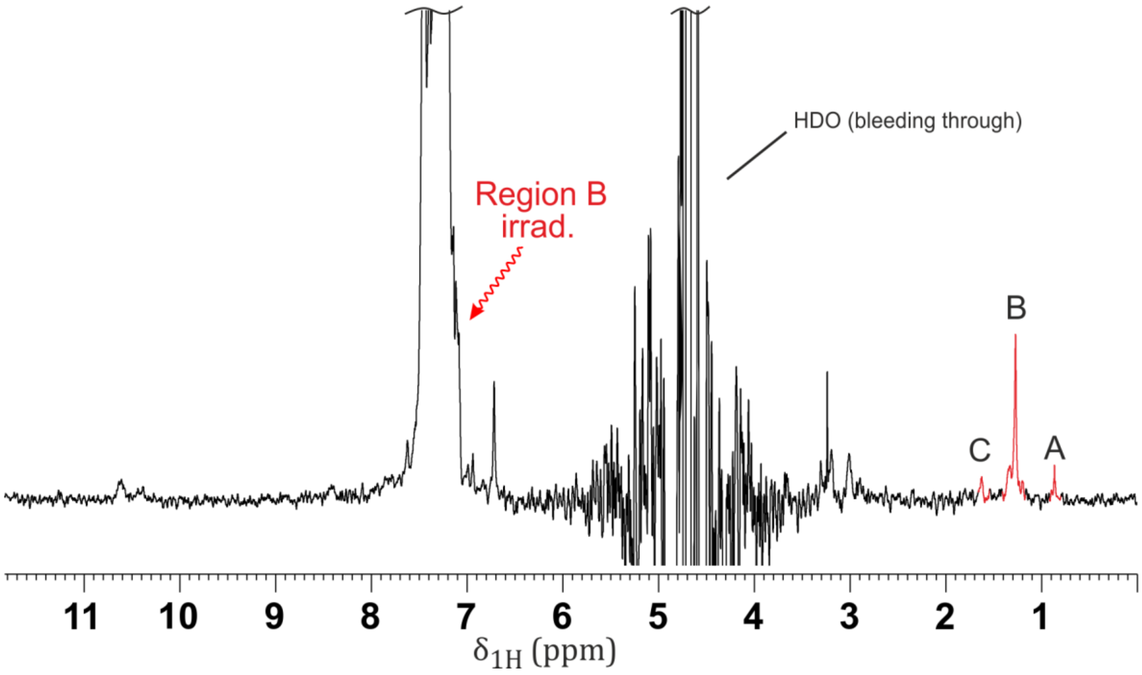

Figure S4D. Selective NOESY

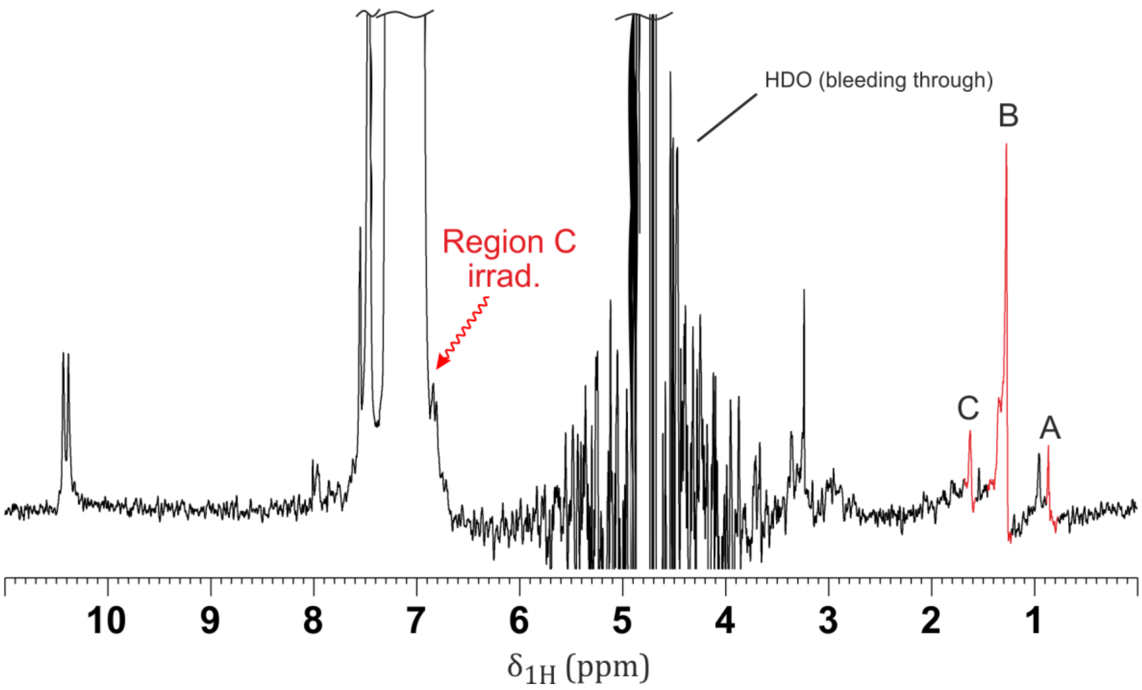

Figure S4E. Selective NOESY

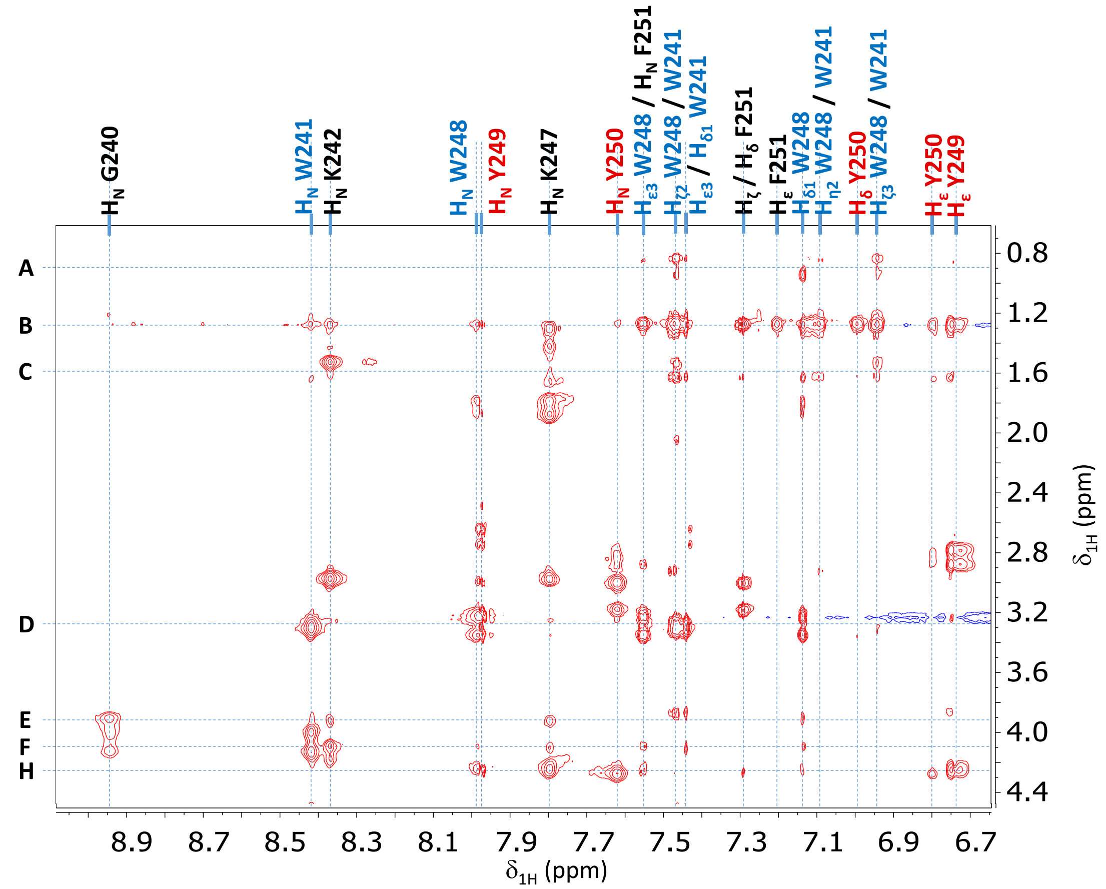

Figure S4F. 2D $^{1}H,^{1}H$-NOESY spectrum of 1 mM $LytA_{239\text{-}252}$ peptide and 30 mM DPC micelles in aqueous solution (H_2O/D_2O 9:1 v/v). The NMR parameters were 3 s of relaxation delay, 32 scans, 16 "dummy" scans, 100 ms of mixing time and 4096 x 256 FID size. Signals labeled in blue and red (on the top of the spectrum) correspond to Trp and Tyr residues, respectively, as they are involved in the interaction with the micelles (signal identification on the left of the spectrum, labeled as A to H on the chemical structure). As shown in the DPC micelle structure, the interaction takes place principally with the lipophilic part of the micelle (B region). Note that certain protons of residues close in the 3D structure to the aromatic side chains of the peptide also interact with the micelles, namely G240, K242, K247 and F251.

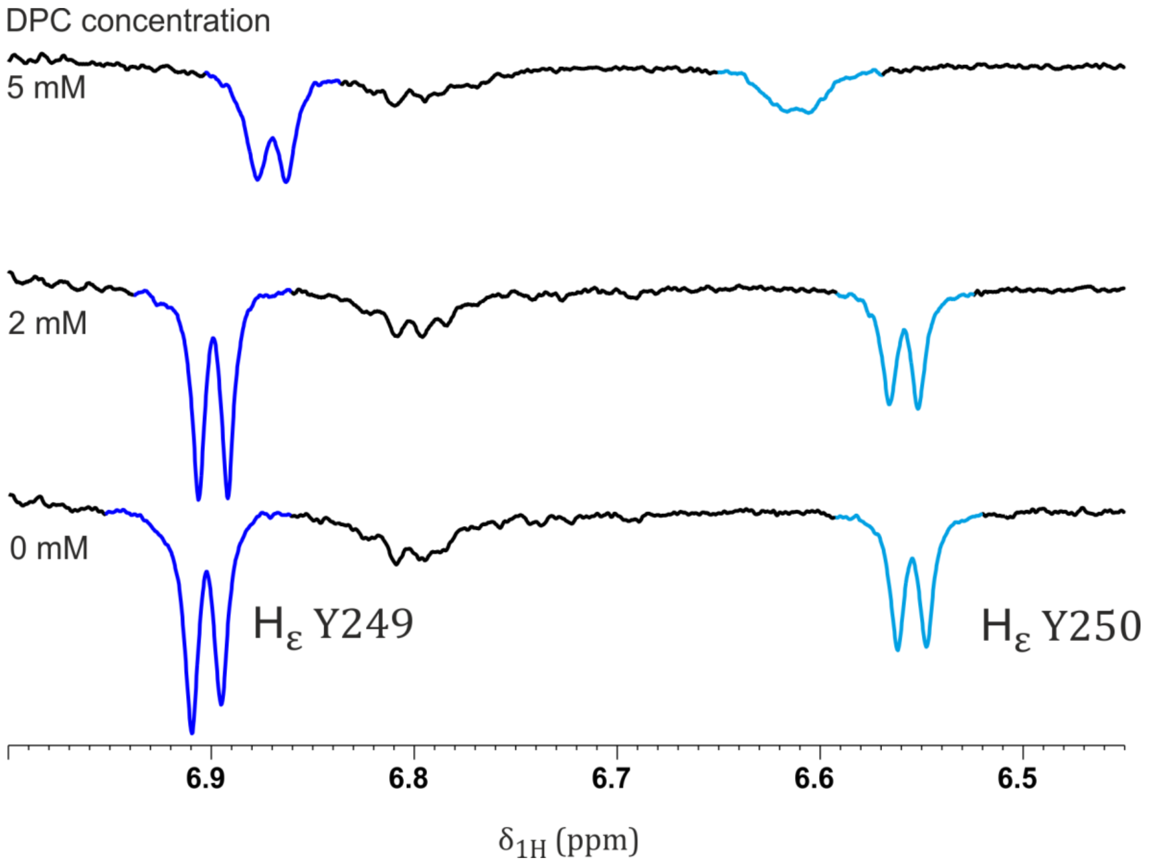

Figure S5. Photo-CIDNP ('light-minus-dark' representation) NMR spectra for DPC micelle titration (from 0 to 5 mM) of 1 mM LytA$_{239\text{-}252}$ peptide for the epsilon protons of Y249 and Y250. The experimental conditions of Method A have been used. 5 mM of DPC induces high line broadening which hampers photo-CIDNP spectroscopic studies. Therefore, the study of the cross-polarization effects which show a different mobility for the two tyrosine residues has been studied up to 2 mM.

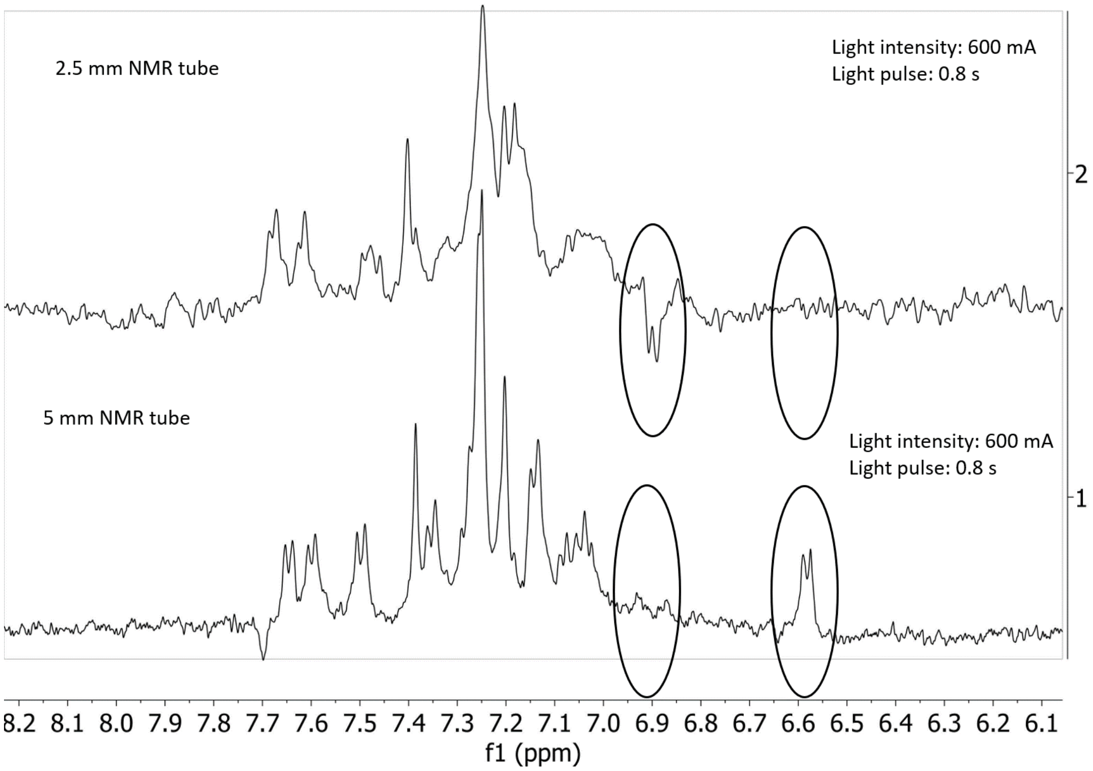

Figure S6. **Optimization of NMR tube diameter for certain experimental conditions (600 mA and 0.8 seconds as light pulse).** Light spectra for 2mM LytA$_{239\text{-}252}$ peptide in a 2.5 mm NMR tube in the absence of DPC micelle. Higher light intensity values for 5 mm NMR tubes did not afforded the expected negative patterns for the tyrosine residues. The tip of the fiber was sandblasted to make it emit light over the whole range following the approach reported by some of us.[23]

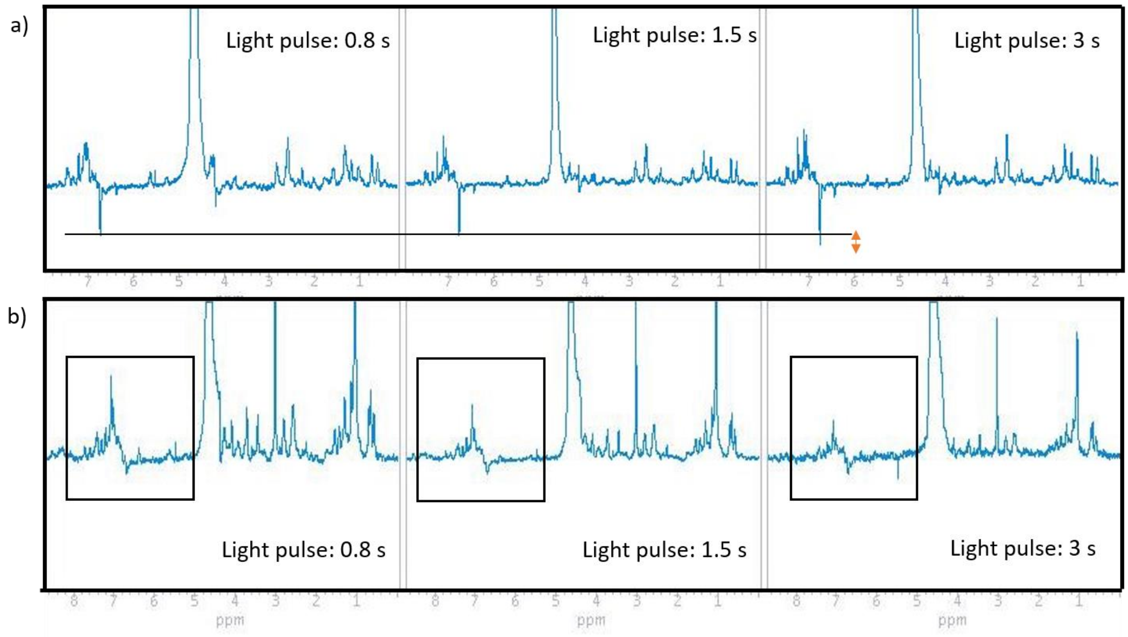

Figure S7. Optimization of the light pulse duration for a light intensity of 600 mA. Light spectra for 2mM LytA$_{239\text{-}252}$ peptide in a 2.5 mm NMR tube. (a) Light NMR spectrum in the absence of DPC. (b) Light NMR spectrum in the presence of DPC. The orange arrow at the horizontal line shown in (a) indicate that the highest signal enhancement for Y249, and therefore the optimum light transmission within the active volume is observed for the longest light pulse. Higher values than 3 s induces signal distortion. The boxes shown in (b) shows that the highest signal-to-noise ratio at the aromatic region, the most informative for us, is observed for 0.8 seconds. Considering that the light pulse for both, absence, and presence of micelle, should be the same, 0.8 seconds was chosen to carry out all experiments in Method B.

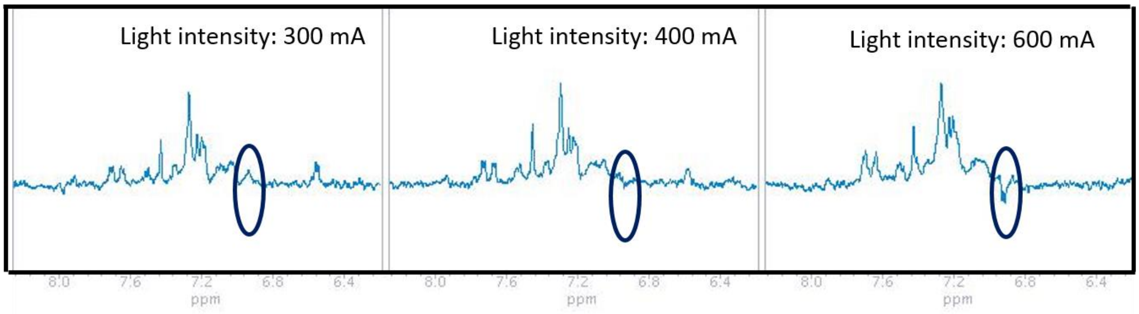

Light pulse: 0.8 s

Figure S8. Optimization of light intensity for an optimized light pulse value. Light spectra for 2mM LytA$_{239\text{-}252}$ peptide in a 2.5 mm NMR tube in the presence of DPC micelle (the most disfavoured conditions). The light setup enabled the use of higher intensity values than 600 mA, however these values induced signal distortions due to sample overheating.

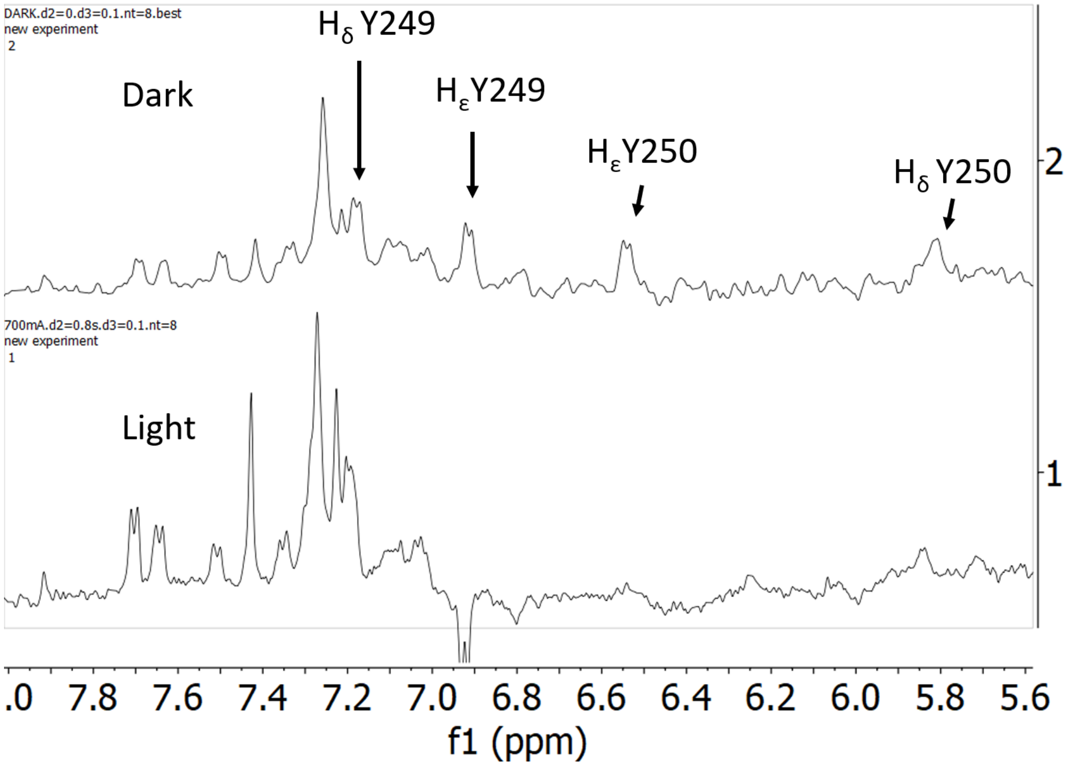

Figure S9. Expansion of the ^{1}H-NMR spectra for 2 mM LyA$_{239\text{-}252}$ for dark (top) and light (bottom) conditions for 0mM of micelle (DPC) (2.5 mm NMR tubes). The spectra were acquired following Method B which enabled higher photo-CIDNP effects due to the lower sample volume (NMR tubes of 2.5 mm) and therefore the more uniform illumination in comparison to 5 mm NMR tubes for the laser diode setup.

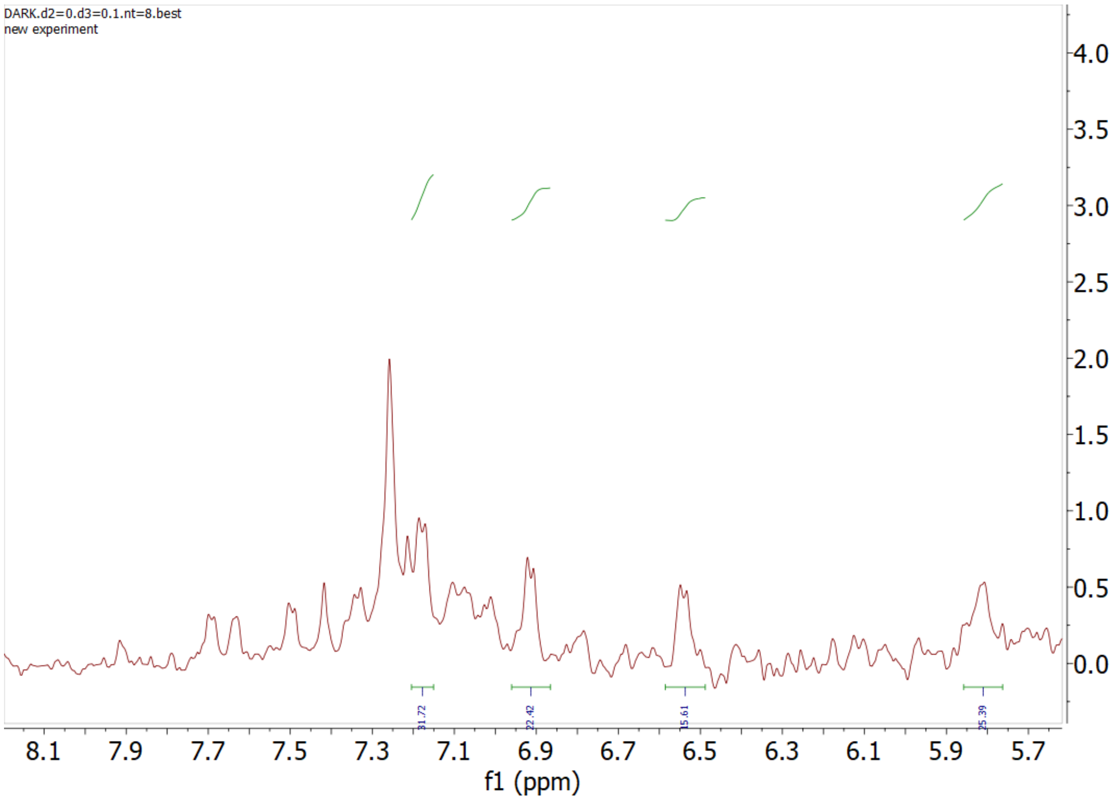

Figure S9A. Expansion of the ^{1}H-NMR spectra for 2 mM LyA$_{239\text{-}252}$ for dark conditions for 0 mM of micelle (DPC) for 2.5 mm NMR tubes. The integral used to calculate the plot shown in Figure 4 are given.

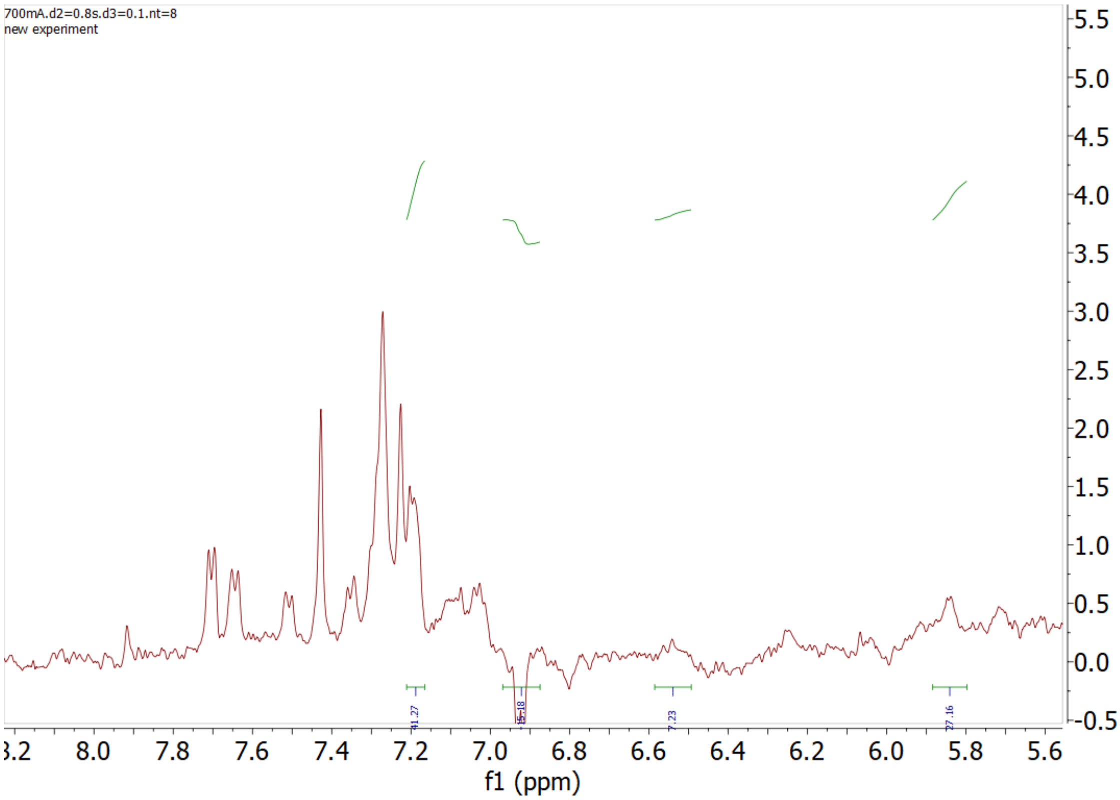

Figure S9B. **Expansion of the ^{1}H-NMR spectra for 2 mM LyA$_{239-252}$ for light conditions for 0mM of micelle (DPC) for 2.5 mm NMT tubes**. The integral used to calculate the plot shown in Figure 4 are given.

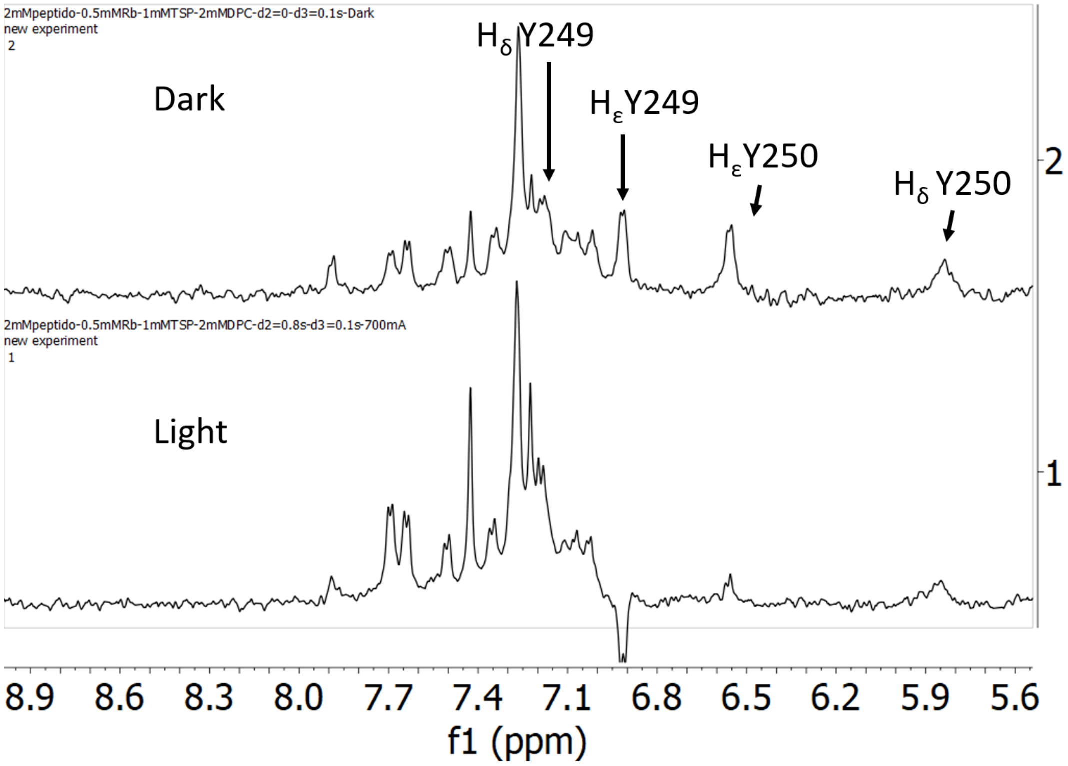

Figure S10. Expansion of the ^{1}H-NMR spectra for 2 mM LyA$_{239\text{-}252}$ for dark (top) and light (bottom) conditions for 2mM of micelle (DPC). The spectra were acquired following Method B which enabled higher photo-CIDNP effects due to the lower sample volume (NMR tubes of 2.5 mm) and therefore the more uniform illumination in comparison to 5 mm NMR tubes.

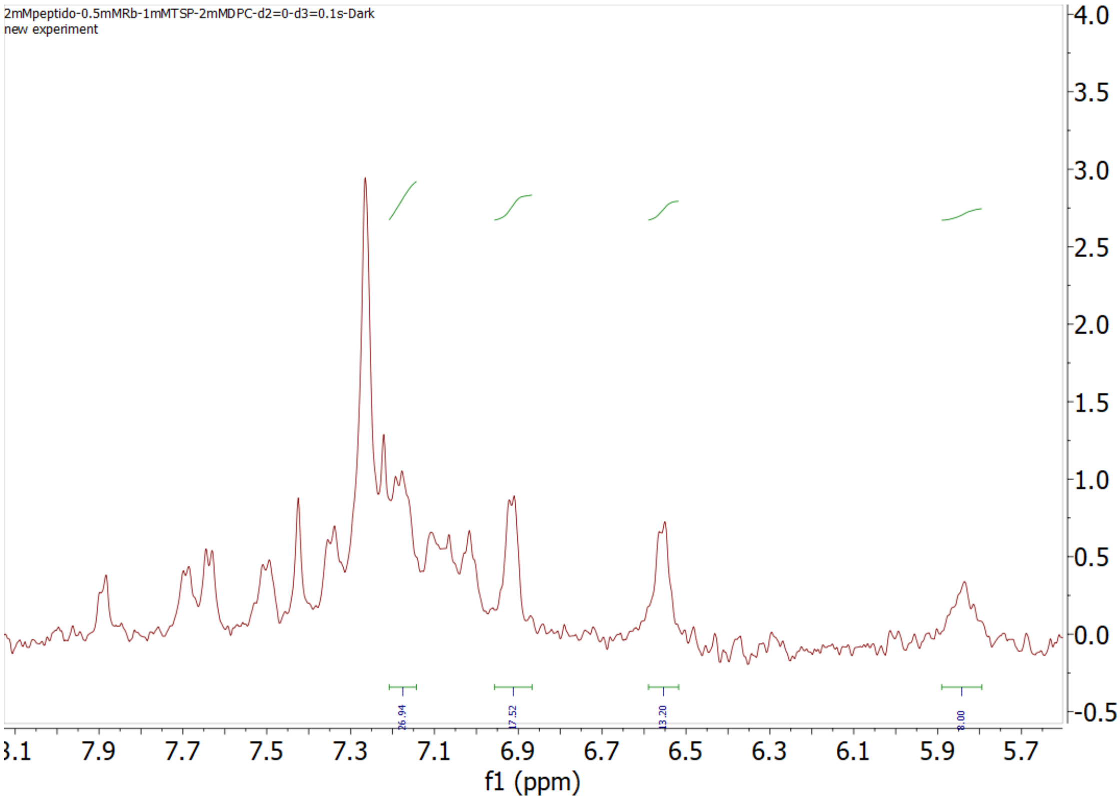

Figure S10A. Expansion of the ^{1}H-NMR spectra for 2 mM LyA$_{239\text{-}252}$ for dark conditions for 2mM of micelle (DPC) for a 2.5 mm NMR tube. The integral used to calculate the plot shown in Figure 4 are given.

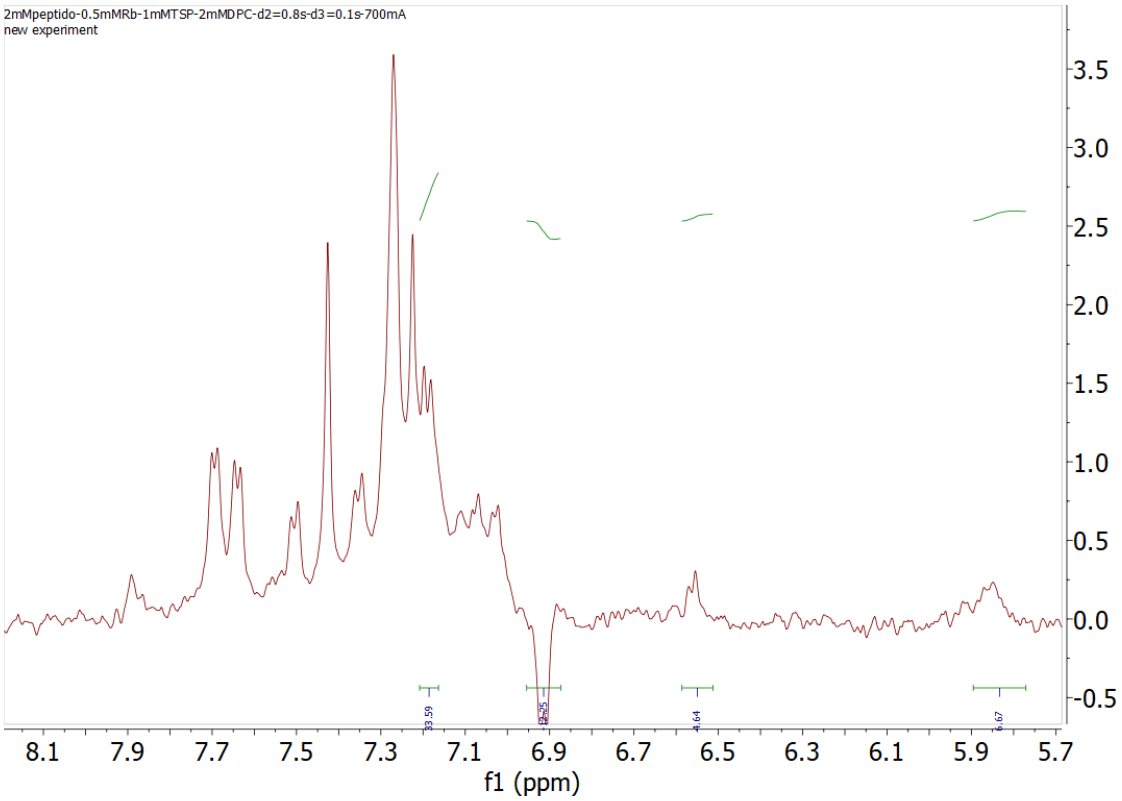

Figure S10B. Expansion of the ^{1}H-NMR spectra for 2 mM LyA$_{239-252}$ for light conditions for 2mM of micelle (DPC) for a 2.5 mm NMR tube. The integral used to calculate the plot shown in Figure 4 are given.
